# Right atrium size in the general population

**DOI:** 10.1038/s41598-021-01968-y

**Published:** 2021-11-18

**Authors:** Karsten Keller, Christoph Sinning, Andreas Schulz, Claus Jünger, Volker H. Schmitt, Omar Hahad, Tanja Zeller, Manfred Beutel, Norbert Pfeiffer, Konstantin Strauch, Stefan Blankenberg, Karl J. Lackner, Jürgen H. Prochaska, Eberhard Schulz, Thomas Münzel, Philipp S. Wild

**Affiliations:** 1 Department of Cardiology, Cardiology I, University Medical Center, Johannes Gutenberg University Mainz, Langenbeckstrasse 1, 55131 Mainz, Germany; 2 Center for Thrombosis and Haemostasis, University Medical Center Mainz, Mainz, Germany; 3 Medical Clinic VII, Department of Sports Medicine, University Hospital Heidelberg, Heidelberg, Germany; 4 Department of General and Interventional Cardiology, University Heart Center Hamburg, Hamburg, Germany; 5 German Center for Cardiovascular Research (DZHK), Partner Site Hamburg/Kiel/Lübeck, Hamburg, Germany; 6 Preventive Cardiology and Preventive Medicine – Department of Cardiology, University Medical Center, Johannes Gutenberg University Mainz, Langenbeckstr. 1, 55131 Mainz, Germany; 7 Department of Psychosomatic Medicine and Psychotherapy, University Medical Center, Johannes Gutenberg University Mainz, Langenbeckstr. 1, 55131 Mainz, Germany; 8 German Center for Cardiovascular Research (DZHK), Partner Site Rhine-Main, Mainz, Germany; 9 Department of Ophthalmology, University Medical Center, Johannes Gutenberg University Mainz, Mainz, Germany; 10 Institute for Medical Biometrics, Epidemiology and Informatics (IMBEI), University Medical Center, Johannes Gutenberg University Mainz, Obere Zahlbacher Str. 69, 55131 Mainz, Germany; 11 Institute of Clinical Chemistry and Laboratory Medicine, University Medical Center, Johannes Gutenberg University Mainz, Langenbeckstr. 1, 55131 Mainz, Germany; 12 Department of Cardiology, Allgemeines Krankenhaus Celle, Celle, Germany

**Keywords:** Cardiology, Medical research, Risk factors

## Abstract

Echocardiography is the most common routine cardiac imaging method. Nevertheless, only few data about sex-specific reference limits for right atrium (RA) dimensions are available. Transthoracic echocardiographic RA measurements were studied in 9511 participants of the Gutenberg-Health-Study. A reference sample of 1942 cardiovascular healthy subjects without chronic obstructive pulmonary disease was defined. We assessed RA dimensions and sex-specific reference limits were defined using the 95th percentile of the reference sample. Results showed sex-specific differences with larger RA dimensions in men that were attenuated by standardization for body-height. RA-volume was 20.2 ml/m in women (5th–95th: 12.7–30.4 ml/m) and 26.1 ml/m in men (5th–95th: 16.0–40.5 ml/m). Multivariable regressions identified body-mass-index (BMI), coronary artery disease (CAD), chronic heart failure (CHF) and atrial fibrillation (AF) as independent key correlates of RA-volume in both sexes. All-cause mortality after median follow-up-period of 10.7 (9.81/11.6) years was higher in individuals who had RA volume/height outside the 95% reference limit (HR 1.70 [95%CI 1.29–2.23], *P* = 0.00014)). Based on a large community-based sample, we present sex-specific reference-values for RA dimensions normalized for height. RA-volume varies with BMI, CHF, CAD and AF in both sexes. Individuals with RA-volume outside the reference limit had a 1.7-fold higher mortality than those within reference limits.

Echocardiography is the dominant imaging modality for cardiac screening and follow-up examinations^1–4^. It is used as a fast and non-invasive tool for distinguishing normal from pathological conditions of cardiac anatomy and function. Therefore, an assessment of standardized, sex- and age-specific reference limits of cardiac structure and function is important^2,5,6^. Despite widespread use of echocardiography worldwide, there are very limited data available regarding dimensions of the right atrium (RA)^2,3,6,7^. Lack of such data from population-based studies may potentially contribute to inaccuracies in sex-specific reference limits for RA dimensions in literature. Moreover, most of the published reference limits are presented as absolute values that were not normalized to body surface area (BSA)^2^ or height^6,8,9^, which is problematic because body size is a key determinant of cardiac measurements.

Therefore, the objective of the present investigation was to analyze RA dimensions from a large-scale, epidemiological study conducted with state-of-the-art echocardiography imaging technology in order to define sex-specific reference limits for the RA.

## Methods

We studied echocardiographic measurements of the RA in 9511 participants of the population-based Gutenberg Health Study (GHS)^10^.

### Study sample

The GHS sample was designed as a population-based, prospective and observational, single-center cohort study in Rhine-Main region of mid-western Germany. Primary aim of the GHS was to characterize novel cardiovascular risk factors (CVRF) in the population.

GHS design as well as inclusion/exclusion criteria have been published previously^5,10^. Briefly summarized, a 1:1 stratification of the study sample for sex (4794 men, 4717 women), residence (urban and rural) and equal strata for age decades was assigned. Enrolled subjects were between 35 and 74 years of age. A written informed consent from each participant was required to take part in the study. The Ethics Committee of the State Chamber of Physicians of Rhineland-Palatinate, Germany (Reference No.: 837.020.07[5555], date of approval: 22.03.2007), and the local and federal data safety commissioners approved the study protocol and the sample design.

Between April 2007 and April 2012, cross-sectional data from 9511 people were acquired and investigated. Overall, 55.5% of the individuals who were invited consented to participate in the study.

Participants were interviewed in a standardized fashion. Measurements of laboratory parameters from venous blood samples, blood pressure, anthropometric data and a transthoracic echocardiography (TTE) were performed. All examinations were carried out according to a standardized protocol by certified medical technical assistants.

### Reference sample

In order to obtain cardiac metric values of healthy participants from the GHS study sample, a subset of subjects without CVRF or history of cardiovascular disease (CVD) as well as right heart strain associated diseases such as chronic obstructive pulmonary disease (COPD) was defined as the reference sample. The reference sample comprised 1942 cardiovascular healthy subjects with no apparent or reported history of chronic heart failure (CHF), myocardial infarction (MI), coronary artery disease (CAD), peripheral artery disease, stroke, arterial hypertension, smoking, dyslipidaemia, obesity (defined as Body-Mass-Index [BMI] > 30 kg/m^2^), diabetes mellitus or family history (FH) of MI/stroke as well as COPD. This group was used to formulate reference values.

### Echocardiography

All participants underwent multimodal TTE with an iE33 echocardiography system and a S5-1 sector array transducer (Royal Philips Electronics, Amsterdam, Netherlands), phased array with 80 elements and a 5- to 1-MHz operating frequency range. Echocardiographic measurements were performed according to current American and European guidelines^2,3,6,9^.

**Figure 1:**
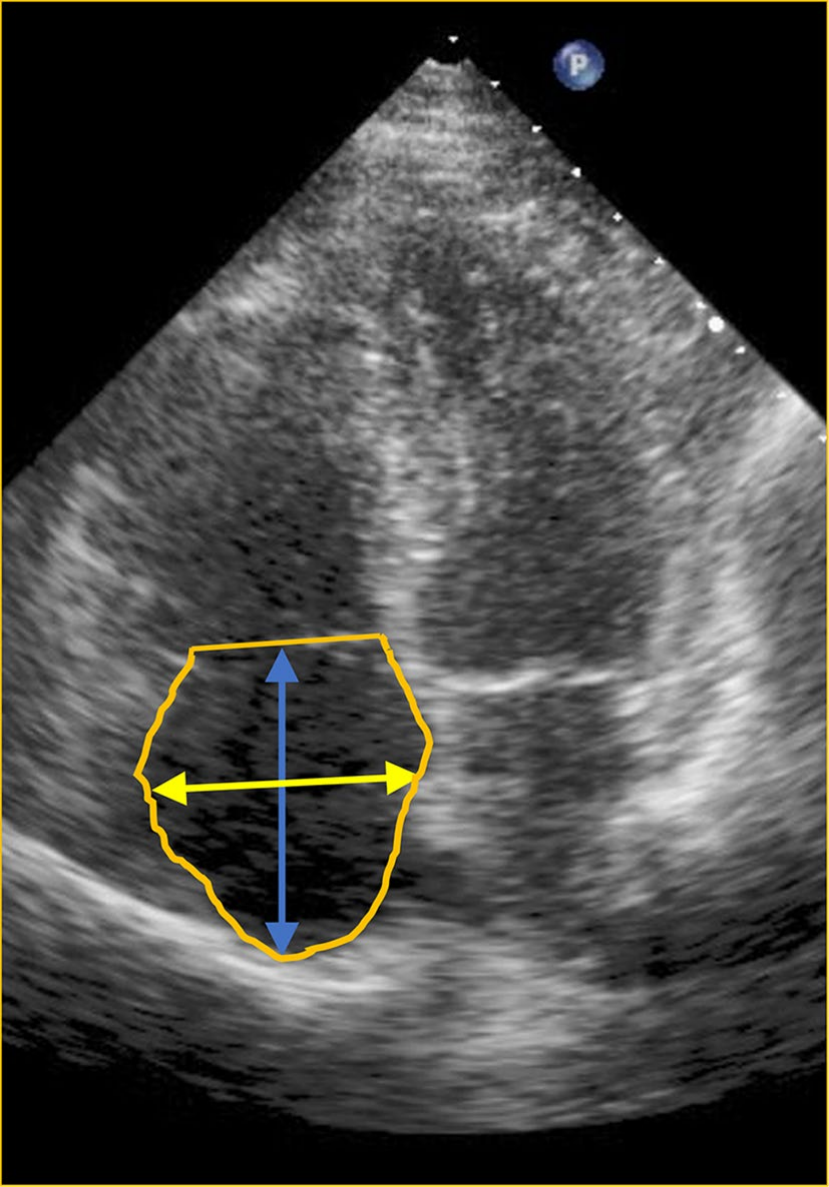
Long axis (apico-basal axis) diameter was measured from RA roof (center of superior RA wall) to the center of tricuspid valve annulus, parallel to interatrial septum (blue arrow), whereas short axis (septal-lateral axis), plane perpendicular to RA long axis was defined that reflects the maximum diameter between the lateral border of the RA and the inter-atrial septum (yellow arrow). Circumference was drawn from lateral to septal border of the tricuspid annulus, excluding the area between tricuspid leaflets and annulus, along RA endocardium, excluding Vena cava inferior/Vena cava superior and RA appendage (yellow line)^2^.

Linear measurements were acquired from 2D images in the apical four-chamber view at ventricular end-systole (maximum RA size). Short and long axis as well as circumference were determined and recorded (Fig. 1), while planimetric area and volume were calculated from these linear measurements. Long axis (apico-basal axis) diameter was measured from RA roof (center of superior RA wall) to the center of tricuspid valve annulus, parallel to interatrial septum. For analysis of the short axis (septal-lateral axis), plane perpendicular to RA long axis was defined that reflects the maximum diameter between the lateral border of the RA and the inter-atrial septum (Fig. 1)^2,3^.

Circumference was drawn from lateral to septal border of the tricuspid annulus, excluding the area between tricuspid leaflets and annulus, along RA endocardium, excluding Vena cava inferior/Vena cava superior and RA appendage (Fig. 1). Circumference value was used to calculate RA area and volume. RA volume was analysed and calculated by the monoplane area-length ellipsoid method. The reproducibility of this method has been previously reported^2,3,7^.

### Data recording and quality control

All digitally recorded datasets (image-management system Xcelera (Royal Philips Electronics, Amsterdam, Netherlands)) were controlled for quality by an experienced echocardiographer. Moreover, data were checked by a central data management unit. Echocardiographic measurements were available in 98.1%, which were included in the final analysis (only 1.9% were missings due patient factors or technical aspects).

### Statistics

Analyses were performed sex-specifically. Data are presented as absolute numbers, percentages, mean with standard deviation or median with 25th and 75th percentiles as appropriate. Distributions of RA measurements were approximately normal. Reference limits, cut-off values for deviation of the reference and percentile values were presented as absolute values and normalized for height and BSA (BSA formula according to Du Bois was used for calculation of the BSA values). Categorization of the reference limits and of the deviation categories of values exceeding the reference limits were defined as followed: the healthy reference comprised all values between the 5th and 95th percentile of the reference sample. Mild deviation contained values between the 95th percentile of the reference sample and the 98th percentile of the entire GHS population sample. Moderate deviation was defined as values between the 98th and 99th percentile of population sample of GHS; and severe deviation included values > 99th percentile of the population sample. A similar strategy has been used by other investigators^5^.

Sex-specific nomograms were generated to correlate RA volume normalized for height and age with defined categories of pathological conditions. Nomograms were generated by quantile regression. We used a multivariable linear regression model to investigate the association between CVRF, co-morbidities and RA dimensions. The model was adjusted for age (10 years), BMI (5 kg/m^2^), diabetes mellitus, dyslipidaemia, arterial hypertension, smoking, and FH of MI.

In addition, Kaplan–Meier plots for cumulative survival were computed to investigate the effect of RA volume (standardized for body height) enlargement on the survival of this large cohort during the median follow-up period of 10.7 (9.81/11.6) years. We calculated a Cox-regression model for all-cause mortality to evaluate the effect of being outside of the 95th percentile of the reference sample in regard to RA volume standardized for height. The multivariate Cox regression model was adjusted for (1) age, sex and CVRF and (2) age (10 years), sex, CVRF, CAD, MI, CHF, atrial fibrillation (AF), stroke, COPD and peripheral artery disease.

Results were not adjusted for multiple testing and might in part be deemed exploratory and warrant replication. As such *P* values < 0.05 were indicative of statistical significance. All analyses were performed with the software R version 2.15 (http://www.R-project.org).

### Ethical standards and informed consent statement

The Gutenberg Health Study (GHS) has been approved by the local ethics committee (Reference No. 837.020.07[5555]) and the data protection officer. The GHS therefore has been performed in accordance with the ethical standards laid down in the 1964 Declaration of Helsinki and its later amendments as well as the recommendations for Good Clinical and Epidemiological Practice. All study participants gave their written informed consent prior to their inclusion in the study.

## Results

Overall, 9511 participants (age range 35–74 years; 4794 men, 4717 women) were enrolled in the GHS study sample to investigate RA metrics. Age of men and women of the study sample was similar (54.8 ± 11.2 vs. 54.6 ± 11.1 years). Prevalence of CVRF except for FH of MI/stroke was higher in men than in women. Accordingly, co-morbidities including MI, CAD, stroke and atrial fibrillation (AF) were encountered more frequently in male than in female participants (Table S1 of supplemental data).

Overall, 20.4% of the 9,511 subjects had no CVD, COPD or CVRF and were included in the healthy reference sample (n = 1942). Women prevailed among this group (61.1% women, 38.9% men). Characteristics of the cardiovascular healthy reference sample are shown in Table S1 of supplemental data.

Absolute RA measurements revealed sex-specific differences with larger values in men. After standardization for body height, these differences were attenuated in the reference sample. Absolute septal-lateral (short axis) and apico-basal (long axis) diameters were 11.2% and 7.6% larger in men than in women. After normalization for height, these differences decreased to 3.7% and 1.8%, respectively. Absolute mean values of RA circumference, area and volume were 10.3%, 19.6% and 28.4% larger in men compared to women. Normalization for height reduced these sex-specific differences to 2.9%, 12.8% and 22.6%, respectively. Values and sex-specific cut-off values for the reference limits of RA diameter, circumference, area and volume are presented in Tables 1 and 2 (values of the overall cohort are shown in Tables S2 and S3 of the supplement and values normalized for body surface area (BSA) are shown in Tables S4 and S5 of the supplement).

**Table 1:** Distribution of right atrial measurements according to sex in a reference sample of subjects without CVRF and CVD (n = 1942): absolute values and values normalized for height.

	Mean	2SD-interval	Median	5th–95th percentile
**Right atrium—absolute values**				
**Men**				
Circumference (cm)	15.4	12.7–18.2	15.3	13.3–17.8
Area (cm^2^)	16.8	10.7–22.8	16.4	12.4–22.5
Volume (ml)	46.9	19.7–74.1	45.0	28.6–73.3
Septal-lateral diameter (cm)	3.84	2.92–4.76	3.80	3.10–4.70
Apico-basal diameter (cm)	4.86	3.85–5.86	4.90	4.10–5.80
**Women**				
Circumference (cm)	13.8	11.4–16.3	13.8	12.0–15.9
Area (cm^2^)	13.5	8.7–18.4	13.3	10.0–17.8
Volume (ml)	33.6	14.6–52.5	32.2	20.7–51.0
Septal-lateral diameter (cm)	3.41	2.65–4.18	3.40	2.80–4.10
Apico-basal diameter (cm)	4.49	3.49–5.49	4.50	3.70–5.30
**Right atrium—values normalized for height**				
**Men**				
Circumference/height (cm/m)	8.60	7.09–10.1	8.55	7.39–9.88
Area/height (cm^2^/m)	9.34	6.09–12.6	9.17	6.93–12.4
Volume/height (ml/m)	26.1	11.4–40.8	25.0	16.0–40.5
Septal-lateral diameter/height (cm/m)	2.14	1.64–2.64	2.11	1.74–2.64
Apico-basal diameter/height (cm/m)	2.71	2.14–3.28	2.69	2.27–3.21
**Women**				
Circumference/height (cm/m)	8.35	6.86–9.84	8.30	7.18–9.69
Area/height (cm^2^/m)	8.14	5.28–11.0	8.01	6.08–10.6
Volume/height (ml/m)	20.2	8.99–31.4	19.4	12.7–30.4
Septal-lateral diameter/height (cm/m)	2.06	1.60–2.51	2.04	1.73–2.47
Apico-basal diameter/height (cm/m)	2.66	2.09–3.23	2.65	2.21–3.14

**Table 2:** Reference limits and categorization of values exceeding the reference limits for variables of the right atrium in the GHS population sample: absolute values and values normalized for height.

	Reference	Mild 95th Ref–98thPctl	Moderate 98th–99thPctl	Severe > 99th–99.9thPctl	Very severe > 99.9thPctl
		Grade of deviation from the reference			
**Right atrium—absolute values**					
**Men**					
Circumference (cm)	≤ 17.8	17.9 < v ≤ 18.7	18.7 < v ≤ 19.2	19.2 < v ≤ 21.0	> 21.0
Area (cm^2^)	≤ 22.5	22.4 < v ≤ 24.3	24.3 < v ≤ 25.5	25.5 < v ≤ 30.4	> 30.4
Volume (ml)	≤ 73.3	72.0 < v ≤ 81.9	81.9 < v ≤ 88.5	88.5 < v ≤ 111	> 111
Septal-lateral diameter (cm)	≤ 4.70	4.60 < v ≤ 4.80	4.80 < v ≤ 5.00	5.00 < v ≤ 5.40	> 5.40
Apico-basal diameter (cm)	≤ 5.80	5.80 < v ≤ 6.10	6.10 < v ≤ 6.30	6.30 < v ≤ 7.00	> 7.00
**Women**					
Circumference (cm)	≤ 15.9	16.4 < v ≤ 17.0	17.0 < v ≤ 17.5	17.5 < v ≤ 19.0	> 19.0
Area (cm^2^)	≤ 17.8	18.7 < v ≤ 20.1	20.1 < v ≤ 21.0	21.0 < v ≤ 25.1	> 25.1
Volume (ml)	≤ 51.0	54.2 < v ≤ 61.4	61.4 < v ≤ 66.1	66.1 < v ≤ 84.5	> 84.5
Septal-lateral diameter (cm)	≤ 4.10	4.10 < v ≤ 4.40	4.40 < v ≤ 4.50	4.50 < v ≤ 5.01	> 5.01
Apico-basal diameter (cm)	≤ 5.20	5.30 < v ≤ 5.60	5.60 < v ≤ 5.80	5.80 < v ≤ 6.40	> 6.40
**Right atrium—values normalized for height**					
**Men**					
Circumference/height (cm/m)	≤ 9.88	10.1 < v ≤ 10.6	10.6 < v ≤ 10.9	10.9 < v ≤ 12.4	> 12.4
Area/height (cm^2^/m)	≤ 12.4	12.5 < v ≤ 13.6	13.6 < v ≤ 14.4	14.4 < v ≤ 17.1	> 17.1
Volume/height (ml/m)	≤ 40.5	40.1 < v ≤ 46.0	46.0 < v ≤ 49.4	49.4 < v ≤ 62.5	> 62.5
Septal-lateral diameter/height (cm/m)	≤ 2.64	2.58 < v ≤ 2.72	2.72 < v ≤ 2.81	2.81 < v ≤ 3.06	> 3.06
Apico-basal diameter/height (cm/m)	≤ 3.21	3.30 < v ≤ 3.46	3.46 < v ≤ 3.58	3.58 < v ≤ 3.93	> 3.93
**Women**					
Circumference/height (cm/m)	≤ 9.69	10.0 < v ≤ 10.5	10.5 < v ≤ 10.8	10.8 < v ≤ 11.9	> 11.9
Area/height (cm^2^/m)	≤ 10.6	11.3 < v ≤ 12.2	12.2 < v ≤ 13.0	13.0 < v ≤ 15.6	> 15.6
Volume/height (ml/m)	≤ 30.4	32.9 < v ≤ 36.9	36.9 < v ≤ 40.0	40.0 < v ≤ 52.1	> 52.1
Septal-lateral diameter/height (cm/m)	≤ 2.47	2.52 < v ≤ 2.65	2.65 < v ≤ 2.74	2.74 < v ≤ 3.10	> 3.10
Apico-basal diameter/height (cm/m)	≤ 3.14	3.29 < v ≤ 3.46	3.46 < v ≤ 3.55	3.55 < v ≤ 4.11	> 4.11

*v* stands for the atrial value measured, *Pctl* for percentile, *95th Ref* for 95th percentile of reference sample.

In the reference sample, median short axis was 2.1 cm/m in men (5th–95th percentiles: 1.7–2.6) and 2.1 cm/m in women (5th–95th: 1.7–2.5). Median long axis was 2.7 cm/m in both sexes (5th–95th women: 2.2–3.1; men: 2.3–3.2).

Calculated RA volume was 20.2 ml/m in women (5th–95th: 12.7–30.4 ml/m) and 26.1 ml/m in men (5th–95th: 16.0–40.5 ml/m). The 95th percentiles of the reference sample were defined as reference limit.

**Figure 2:**
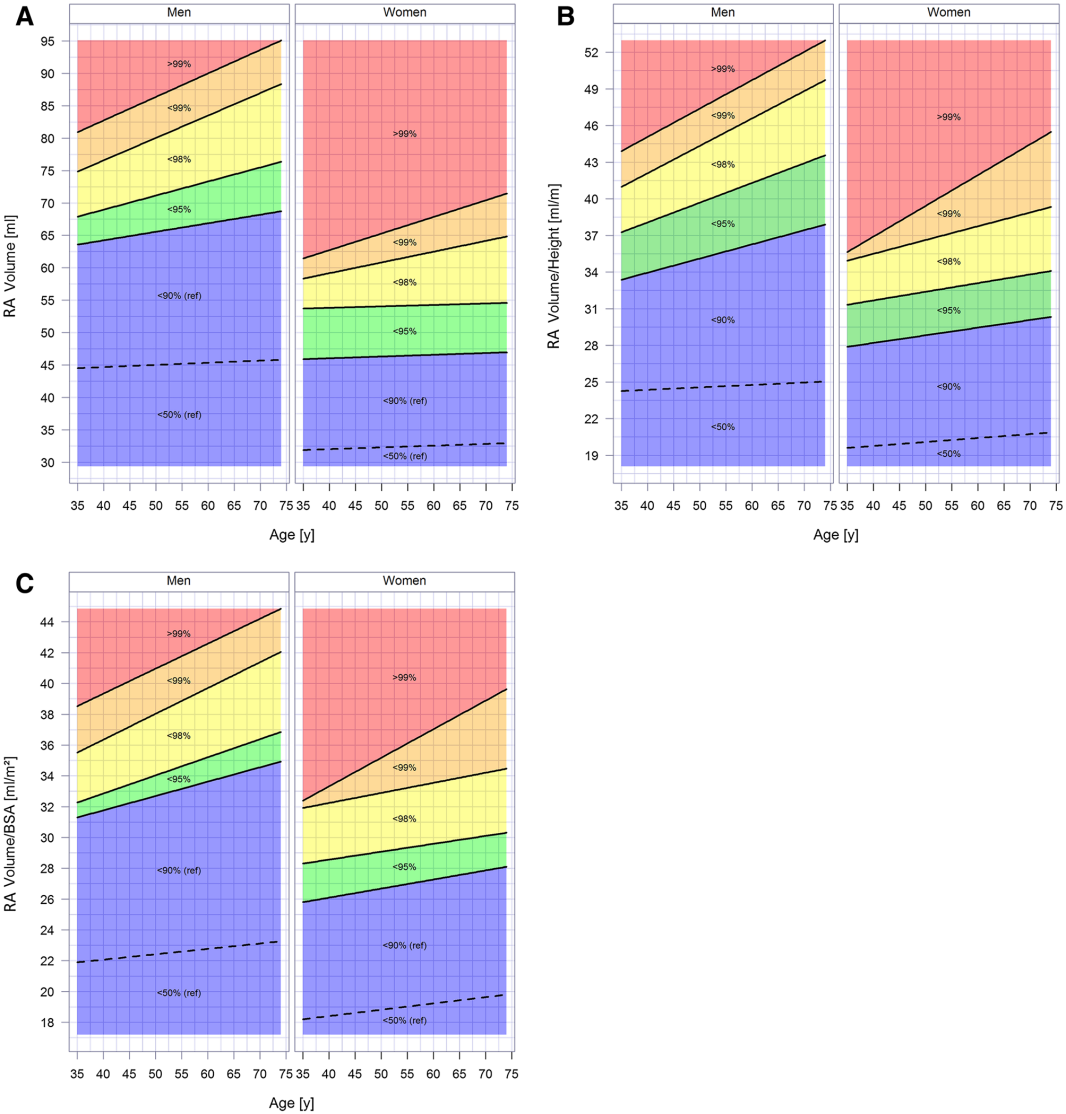
Sex-specific nomogram for volume (**A**), volume/height (**B**) and volume/body surface area (**C**) of the right atrium stratified by age. The lines mark the 95% percentile of reference sample (< 95%) and 98th and 99th percentiles of the GHS population sample. < 95% of reference sample is the normal reference (green and blue areas). Mild deviation is marked in yellow, severe deviation in orange and very severe deviation in red.

Figure 2 shows a nomogram for correlation of RA volume/height to age in order to define age-dependent and sex-specific reference values and deviation from the cardiovascular healthy subgroup. Nomograms for the other RA measurements could be seen in Figs. S1–S4 of the supplement.

Changes in echocardiographic RA dimensions according to age were small and are shown in Table S6 of the supplement.

Multivariable regression analysis determined age, arterial hypertension, dyslipidaemia and BMI as independently associated with RA volume in men; in women, diabetes mellitus, BMI and dyslipidaemia remained independently associated (Table 3). CHF, CAD and AF were associated with larger RA volumes in both sexes, while history of MI was associated with larger RA volume in men only and COPD with smaller volume in women only (Table 3). After additional adjustment for the use of antihypertensive medication, arterial hypertension remained associated with a smaller RA volume (normalized for height) in men (β − 1.83 [95%CI − 2.41 to − 1.25]; *P* < 0.0001).

**Table 3:** Sex-specific association in GHS study sample between right atrial measurements and classical CVRF and cardiovascular diseases in uni- and multivariable linear regression models.

RA-volume/height (ml/m)	Crude β (95%CI)	*p* value (crude)	Adj. β* (95%CI)	*p* value*
**Men**				
Age (10 years)	**0.41 (0.21–0.60)**	**< 0.0001**	**0.48 (0.27–0.69)**	**< 0.0001**
BMI (5 kg/m^2^)	**1.48 (1.23–1.72)**	**< 0.0001**	**1.66 (1.40–1.93)**	**< 0.0001**
Diabetes mellitus	**1.10 (0.40–1.80)**	**0.0021**	0.19 (− 0.54 to 0.92)	0.62
Dyslipidemia	0.04 (− 0.40 to 0.49)	0.85	− **0.56 (**− **1.01–**− **0.10)**	**0.016**
Family history of MI or stroke	− 0.09 (− 0.64 to 0.47)	0.76	− 0.10 (− 0.65 to 0.46)	0.74
Arterial hypertension	− 0.08 (− 0.52 to 0.36)	0.71	− **1.30 (**− **1.78 to **− **0.82)**	**< 0.0001**
Smoking	− **0.64 (**− **1.18 to **− **0.10)**	**0.021**	− 0.41 (− 0.95 to 0.46)	0.74
Coronary artery disease	**2.62 (1.71 to 3.53)**	**< 0.0001**	**2.36 (1.42–3.30)**	**< 0.0001**
History of MI	**2.14 (1.08–3.20)**	**< 0.0001**	**1.51 (0.43–2.59)**	**0.0060**
Chronic heart failure	**4.46 (2.44–6.48)**	**< 0.0001**	**3.85 (1.84–5.85)**	**0.00017**
History of Stroke	1.19 (− 0.23 to 2.61)	0.10	1.04 (− 0.37 to 2.45)	0.15
COPD	− 0.50 (− 1.59 to 0.59)	0.37	− 0.72 (− 1.80 to 0.36)	0.19
Atrial fibrillation	**7.27 (6.10–8.43)**	**< 0.0001**	**6.88 (5.72–8.04)**	**< 0.0001**
Peripheral artery disease	0.74 (− 0.47 to 1.95)	0.23	− 0.07 (− 1.28 to 1.13)	0.91
**Women**				
Age (10 years)	**0.42 (0.26–0.58)**	**< 0.0001**	0.16 (− 0.01 to 0.34)	0.071
BMI (5 kg/m^2^)	**1.63 (1.49–1.78)**	**< 0.0001**	**1.71 (1.55–1.87)**	**< 0.0001**
Diabetes mellitus	**0.91 (0.21–1.61)**	**0.011**	− 1.02 (− 1.73 to − 0.31)	**0.0048**
Dyslipidemia	**0.41 (0.003–0.82)**	**0.048**	− **0.45 (**− **0.87 to **− **0.03)**	**0.036**
Family history of MI or stroke	0.10 (− 0.32 to 0.51)	0.66	− 0.29 (− 0.69 to 0.12)	0.16
Arterial hypertension	**1.33 (0.98–1.69)**	**< 0.0001**	0.008 (− 0.40 to 0.41)	0.97
Smoking	1.95 (1.56–2.34)	**< 0.0001**	0.07 (− 0.37 to 0.51)	0.76
Coronary artery disease	**2.37 (1.10–3.63)**	**0.00024**	**1.55 (0.31–2.79)**	**0.015**
History of MI	**1.76 (0.19–3.33)**	**0.028**	0.78 (− 0.73 to 2.29)	0.31
Chronic heart failure	**3.09 (1.44–4.74)**	**0.00025**	**2.09 (0.51–3.67)**	**0.0097**
History of stroke	0.25 (− 1.38 to 1.87)	0.77	− 0.20 (− 1.78 to 1.38)	0.80
COPD	− 0.58 (− 1.32 to 0.16)	0.12	− **1.23 (**− **1.95 to **− **0.52)**	**0.00075**
Atrial fibrillation	**6.41 (5.12–7.71)**	**< 0.0001**	**5.57 (4.31–6.82)**	**< 0.0001**
Peripheral artery disease	0.86 (− 0.25 to 1.97)	0.13	− 0.11 (− 1.18 to 0.96)	0.84

β stands for β-estimate.

*In the multivariable linear regression models all classical risk factors except sex listed in this table were included.

*p* values < 0.05 were indicative of statistical significance.

The subjects included in the study were continuously observed for a median period of 10.7 (9.81/11.6) years. In this follow-up period 658 (6.9%) of the individuals died (men: 423 [4.4%], women: 235 [2.5%]).

**Figure 3:**
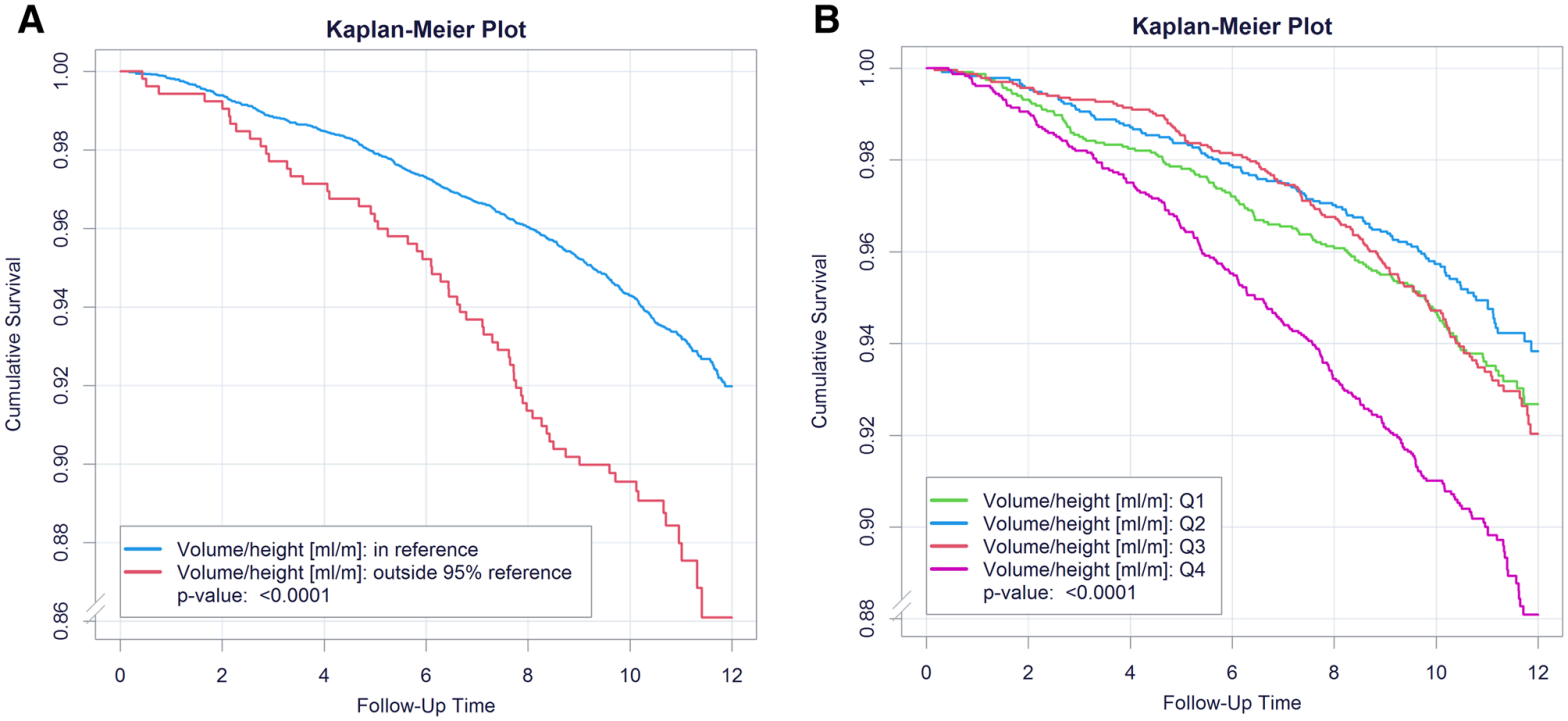
Kaplan–Meier plots for cumulative survival. (**A**) Subjects were subdivided in subjects with right atrial volume (indexed for body height) in the normal range (95th percentile of reference sample) and those outside the reference limit. Groups differed significantly in all-cause mortality with *P* < 0.0001. (**B**) Subjects were subdivided in quartiles by size of right atrial volume (standardized for body height). The quartiles differed significantly with *P* < 0.0001.

Kaplan–Meier plots for cumulative survival showed a better survival for subjects with RA volume (standardized for height) within the 95^th^ percentile of the reference sample (Fig. 3a) as well as within quartiles with lower RA volumes (standardized for height) (Fig. 3b). In the Cox regression models, an individual’s condition of being outside of the 95^th^ percentile of the reference sample in regard to RA volume (indexed for height) at baseline was significantly and independently associated with higher all-cause mortality during the follow-up-period of 10.7 (9.81/11.6) years (adjusted for age, sex and CVRF: HR 1.82 [95%CI 1.40–2.37], *P* < 0.0001; adjusted for age, sex, CVRF, CAD, history of MI, CHF, history of stroke, COPD, AF and PAD: HR 1.70 [95%CI 1.29–2.23], *P* = 0.00014).

## Discussion

The present epidemiological studies established echocardiographic parameters in more than 9,500 participants together with information concerning anthropometric data, laboratory results, disease history and CVRF. The definition of RA reference values was based on a healthy reference group of nearly 2,000 participants without CVRF, right heart strain due COPD or known CVD. Absolute RA measurements revealed sex-specific differences with larger values in men compared to women. After standardization for body height, these sex-specific differences were significantly reduced but remained significant.

To our knowledge, the present investigation is the largest systematic assessment to define reliable sex-specific reference limits of RA long and minor axis, area, circumference and volume that enables us to classify categories of deviation from the healthy reference accurately^1–3,6,8,9,11^. Additionally, normalization for height was used for all measured parameters. Indexation of echocardiographic measurements is a particular area of interest in cardiovascular imaging methologies, since anthropometric differences influence cardiac measurements^12–14^. Absolute values have the limitation that cardiac geometry greatly varies according to body size. Therefore, echocardiographic data should be normalized to anthropometric parameters. Most published reference limits are presented as absolute values or were normalized to BSA^1,2^. However, it has been shown that normalization for BSA may overestimate cardiac dimensions in young individuals and underestimate those in older people^15^. In addition, obesity leads to misclassification of cardiac metrics normalized to BSA^16^. Normalization for BMI or for body weight is both problematic since obesity is a CVRF on its own. This is one cause, why obese people with a BMI above 30 were not included in the healthy reference group in our study. Person’s height has the advantage that it can easily be measured and is not associated with CVD. Because RA reference limits normalized to height were not published so far, we also presented results as absolute values or normalized to BSA in the supplementary to make our values comparable to previous studies.

The results of our study showed distinct sex-specific differences with respect to absolute RA values. However, these sex-related differences were no longer found upon normalization for height. Comparison of our results with other studies is challenging, given the paucity of sex-specific data on RA measurements.

Presented data of absolute short axis RA diameter with mean values of 3.8 cm for men and 3.4 cm for women were close to data of other studies^1,11,17^. Sex-unspecific RA short axis reference range was reported as between 2.9 and 4.5 cm^18^ and 4.4 cm was described as the cut-off value from normal to enlargement^9^. In this present study sex-specific cut-off values of RA short axis from healthy to pathologic were 4.7 cm in men and 4.1 cm in women. Lang et al. described sex-specific reference values indexed for BSA close to the results of our study^2^.

For RA long axis, less data is available. Absolute RA long axis mean values in the present study of 4.9 cm in males and 4.5 cm in females are both larger than the values reported in literature^1,17^. Rudzki et al. defined a RA long axis diameter of > 5.3 cm as pathological^9^. Upper cut-off values in our study were 5.8 cm in men and 5.2 cm in women. Kou et al. reported absolute sex-specific values and Lang et al. sex-specific reference values indexed for BSA both close to our results^1,2^.

Up to now, there are only limited published data available regarding RA circumference and RA area. Results of our study showed slightly larger RA area values than the NORRE study^1^. However, the NORRE study is not a population-based study and presented only absolute values and results normalized for BSA, with the well-known problems described above^1^.

In contrast to available literature, our study found higher absolute sex-specific RA mean volume values of 46.9 ml in men and 33.6 ml in women^1^. Lang et al. reported RA volumes normalized for BSA of 21 ml/m^2^ in women and 25 ml/m^2^ in men, which were similar to our results after indexing for BSA (Table S5 of supplemental data)^2^. In a study of 159 healthy participants, reference values of 18–50 ml/m^2^ for males and of 17–41 ml/m^2^ for females were reported^8^, which showed higher upper reference limits than our study (≤ 36.1 ml/m^2^ in men and ≤ 28.7 ml/m^2^ in women). In contrast to our study, only a smaller number of participants were enrolled and their healthy reference group also included obese subjects up to a BMI of 35 kg/m^2^ as well as participants with a screening blood pressure up to 160/90 mmHg^8^. Our exclusion criteria were more strict with BMI > 30 kg/m^2^ and blood pressure > 139/89 mmHg considering pathological. Therefore, these differences in the exclusion criteria for the cardiovascular reference group may explain the differences regarding cut-off values.

The presented nomogram is analyzed for simple and quick relation between measured RA volume/linear dimensions and age as well as sex.

However, height as indexation also has problems, particularly as it violates geometric assumptions with the theory of similarity (e. g. indexing a 3-dimensional measurement such as volume against a one-dimension parameter such as height).

Identified causes of abnormal RA dimensions in our reference sample are in accordance with some^7,8,11^, but not all studies^8,19^. In the present study a higher BMI, CAD, CHF and AF were independently associated with an increase of RA volume. Arterial hypertension was associated with a smaller RA volume in males, whereas COPD in females.

RA enlargement was described for several diseases such as chronic pulmonary hypertension, pulmonary embolism (PE), COPD, dilated cardiomyopathy, right atrial myocardial infarction and AF^7,8,17,20–26^. With respect to CHF as well as AF, the results of our study are in accordance with the available literature showing RA dilation, while arterial hypertension and COPD did not (as expected) induce RA enlargement in our cohort. Relevance of RA enlargement for survival was emphasized in a Cox regression model. RA enlargement outside the reference limit was associated with approximately 1.7-fold increase of all-cause mortality during median follow-up period of more than 10.5 years. Several pathomechanisms might contribute to this higher mortality rate: the right heart plays a central role for morbidity and mortality of patients with cardiopulmonary diseases^9,25^. RV dysfunction predicts a reduced exercise capacity, autonomic dysbalance and poorer prognosis^25,27^. After acute cardiovascular events such as MI and PE, RV dysfunction is associated with higher mortality and development of heart failure^9,25,27,28^. RA is directly involved in these pathophysiological processes^20,22^. During ventricular diastole, the RA is exposed directly to the RV pressure through the opened tricuspid valve^7^. RA size therefore depends on RV filling pressures and RA enlargement is generally a manifestation of high RA pressure due to a (functional) tricuspid regurgitation or an elevated RV diastolic pressure^7,21,29,30^. RA enlargement may contribute to a higher risk of thromboembolic events such as PE^31,32^. In patients with chronic pulmonary hypertension a RA enlargement is one of the independent predictors for adverse outcome^9,21,22,25,29,33^. Additionally, RA size is a predictor for preservation of sinus-rhythm after intra-operative AF ablation^24^ and in AF patients a decreased RA dilation could be observed a few months after successful cardioversion^23,26^. Overall, there is evidence that remodelling and regression of RA size are associated with improved cardiovascular outcome^34^.

Although 3-dimentional (3-D) echocardiographic might revolutionize RA measurement^35–37^, since RA anatomical structure is complicated, 2D parameters could be easily measured and are an important part of echocardiographic assessment to get a fast and simple impression of RA deviation. Thus, RA evaluation should be incorporated in echocardiographic standard examination and well defined RA reference limits are an indispensable basis for future investigations.

### Limitations

There are some limitations on this study. RA volume is calculated by using 2D parameters (diameters, RA area) and difficult anatomy of the RA may disturb these calculations. Therefore, additional methods to assess RA volumes in such people should be investigated. Further, only 35–75 years aged individuals of German nationality were included. Thus, these data may not be extrapolated to other populations with different ethnic/racial background or other age groups.

## Conclusions

Based on a large population-based study sample, our data present sex-specific reference values for cardiac dimensions of the RA. RA volume varies with BMI and pathological conditions that affect RA size in both sexes include CAD, CHF and AF. Individuals with RA volume outside the reference limit at baseline had an approximately 1.7-fold higher long-term mortality than those subjects within the reference limits.

## Supplementary Information

Supplementary Information.

## Abbreviations

AF: Atrial fibrillation
BMI: Body mass index
BSA: Body surface area
CAD: Coronary artery disease
CHF: Chronic heart failure
COPD: Chronic obstructive pulmonary disease
CVD: Cardiovascular diseases
CVRF: Cardiovascular risk factors
FH: Family history
GHS: Gutenberg Health Study
MI: Myocardial infarction
PE: Pulmonary embolism
RA: Right atrium
RV: Right ventricle
TTE: Transthoracic echocardiography
