# Supplementary material for: Right atrium size in the general population
Source: Sci Rep. 2021 Nov 18;11:22523. doi: 10.1038/s41598-021-01968-y (PMC8602329; doi:10.1038/s41598-021-01968-y)

**Supplemental Material**

**Right atrium size in the general population**

**Keller et al.: Right atrial Size**

Karsten Keller^1,2,3^, Christoph Sinning^4,5^, Andreas Schulz^6^, Claus Jünger^7^, Volker H. Schmitt^1,8^, Omar Hahad^1,8^, Tanja Zeller^4,5^, Manfred Beutel^7^, Norbert Pfeiffer^9^, Konstantin Strauch^10^, Stefan Blankenberg^4,5^, Karl J. Lackner^11,8^, Jürgen H. Prochaska^1,2,6,8^, Eberhard Schulz^1,12^, Thomas Münzel^1,8*^, Philipp S. Wild^1,2,6,8*^

^1^ Department of Cardiology, Cardiology I, University Medical Center, Johannes Gutenberg University Mainz, Mainz, Germany

^2^ Center for Thrombosis and Haemostasis, University Medical Center Mainz, Germany

^3^ Medical Clinic VII, Department of Sports Medicine, University Hospital Heidelberg, Heidelberg, Germany

^4^ Department of General and Interventional Cardiology, University Heart Center Hamburg, Hamburg, Germany

^5^ German Center for Cardiovascular Research (DZHK), Partner Site Hamburg/Kiel/Lübeck,

^6^ Preventive Cardiology and Preventive Medicine – Department of Cardiology, University Medical Center, Johannes Gutenberg University Mainz, Langenbeckstr. 1, 55131 Mainz, Germany

^7^ Department of Psychosomatic Medicine and Psychotherapy, University Medical Center, Johannes Gutenberg University Mainz, Langenbeckstr. 1, 55131 Mainz, Germany

^8^ German Center for Cardiovascular Research (DZHK), Partner Site Rhine-Main, Mainz, Germany

^9^ Department of Ophthalmology, University Medical Center, Johannes Gutenberg University Mainz, Mainz, Germany

^10^ Institute for Medical Biometrics, Epidemiology and Informatics (IMBEI), University Medical Center, Johannes Gutenberg University Mainz, Obere Zahlbacher Str. 69, 55131 Mainz, Germany;

^11^Institute of Clinical Chemistry and Laboratory Medicine, University Medical Center, Johannes Gutenberg University Mainz, Langenbeckstr. 1, 55131 Mainz, Germany

^12^ Department of Cardiology, Allgemeines Krankenhaus Celle, Celle, Germany

*contributed equally.

**Table S1 of Supplement.** Characteristics of the community overall sample (n=9,511) and the reference sample (n=1,942).

Data presented are the absolute and relative frequency of patients and mean ± standard deviation.

| **GHS Sample characteristics** | **GHS population sample**  **(n = 9,511)** | |  | **Reference sample**  **(n = 1,942)** | |
| --- | --- | --- | --- | --- | --- |
|  | **Men** | **Women** |  | **Men** | **Women** |
| Subjects (% (n)) | 50.4 (4,794) | 49.6 (4,717) |  | 38.9 (756) | 61.1 (1,186) |
| Age (years) | 54.8±11.2 | 54.6±11.1 |  | 48.8±10.1 | 48.6±9.8 |
| Height (m) | 1.77±0.07 | 1.64±0.07 |  | 1.79±0.07 | 1.66±0.06 |
| Weight (kg) | 87.7±14.8 | 72.2±15.4 |  | 80.4±9.4 | 64.5±8.7 |
| Body mass index (kg/m^2^) | 28.0±4.4 | 27.0±5.8 |  | 25.0±2.4 | 23.4±2.8 |
| **Cardiovascular risk factors** |  |  |  |  |  |
| Hypertension (% (n)) | 53.5 (2,562) | 43.6 (2,055) |  | 0 | 0 |
| Diabetes (% (n)) | 11.0 (529) | 7.0 (328) |  | 0 | 0 |
| Smoking (% (n)) | 20.9 (998) | 18.3 (863) |  | 0 | 0 |
| Dyslipidemia (% (n)) | 42.8 (2,046) | 25.6 (1,200) |  | 0 | 0 |
| Obesity (% (n)) | 26.7 (1,277) | 24.9 (1,176) |  | 0 | 0 |
| Family history of MI or stroke (% (n)) | 19.3 (923) | 23.8 (1,121) |  | 0 | 0 |
| **Selected cardiovascular diseases** |  |  |  |  |  |
| Coronary artery disease (% (n)) | 6.2 (295) | 2.0 (95) |  | 0 | 0 |
| History of MI (% (n)) | 4.5 (215) | 1.3 (62) |  | 0 | 0 |
| Chronic heart failure (% (n)) | 1.2 (56) | 1.2 (55) |  | 0 | 0 |

n stands for the absolute number of subjects.

**Table S2 of Supplement.** Distribution of right atrium according to sex in the population sample of GHS (n=9,511): absolute values.

Data presented are mean, the twofold standard deviation interval, the median and the 5^th^ to 95^th^ percentile interval.

| **Right atrium** | **Mean** | **2SD-Interval** | **Median** | **5^th^-95^th^**  **Percentile** |
| --- | --- | --- | --- | --- |
| **Men** |  | | | |
| Circumference (cm) | 15.4 | 12.5-18.3 | 15.3 | 13.1-17.9 |
| Area (cm²) | 16.6 | 10.2-23.0 | 16.3 | 11.9-22.4 |
| Volume (ml) | 45.5 | 17.6-73.4 | 43.6 | 26.7-72.0 |
| Septal-lateral diameter (cm) | 3.76 | 2.83-4.69 | 3.70 | 3.00-4.60 |
| Apico-basal diameter (cm) | 4.88 | 3.78-5.99 | 4.90 | 4.00-5.80 |
| **Women** |  | | | |
| Circumference (cm) | 14.1 | 11.4-16.7 | 14.0 | 12.1-16.4 |
| Area (cm²) | 13.9 | 8.67-19.1 | 13.6 | 10.2-18.7 |
| Volume (ml) | 34.8 | 14.3–55.4 | 33.2 | 21.3-54.2 |
| Septal-lateral diameter (cm) | 3.43 | 2.65-4.22 | 3.40 | 2.90-4.10 |
| Apico-basal diameter (cm) | 4.49 | 3.49-5.49 | 4.50 | 3.70-5.30 |

Data presented are mean, the twofold standard deviation interval, the median and the 5^th^ to 95^th^ percentile interval.

**Table S3 of Supplement.** Distribution of right atrium according to sex in the population sample of GHS (n=9,511): values normalized for height.

Data presented are mean, the twofold standard deviation interval, the median and the 5^th^ to 95^th^ percentile interval.

| **Right atrium** | **Mean** | **2SD-Interval** | **Median** | **5^th^-95^th^**  **Percentile** |
| --- | --- | --- | --- | --- |
| **Men** |  | | | |
| Circumference/Height (cm/m) | 8.70 | 7.06-10.3 | 8.66 | 7.42-10.1 |
| Area/Height (cm²/m) | 9.37 | 5.86-12.9 | 9.20 | 6.81-12.5 |
| Volume/Height (ml/m) | 25.6 | 10.3-41.0 | 24.6 | 15.2–40.1 |
| Septal-lateral diameter/Height (cm/m) | 2.12 | 1.61-2.64 | 2.11 | 1.73-2.58 |
| Apico-basal diameter/Height (cm/m) | 2.76 | 2.13-3.39 | 2.74 | 2.27-3.30 |
| **Women** |  | | | |
| Circumference/Height (cm/m) | 8.61 | 6.96-10.3 | 8.55 | 7.35-10.0 |
| Area/Height (cm²/m) | 8.49 | 5.35-11.6 | 8.31 | 6.26-11.3 |
| Volume/Height (ml/m) | 21.2 | 8.93-33.6 | 20.2 | 13.2–32.9 |
| Septal-lateral diameter/Height (cm/m) | 2.10 | 1.62-2.57 | 2.08 | 1.75-2.52 |
| Apico-basal diameter/Height (cm/m) | 2.74 | 2.12-3.37 | 2.73 | 2.26-3.29 |

Data presented are mean, the twofold standard deviation interval, the median and the 5^th^ to 95^th^ percentile interval.

**Table S4 of Supplement.** Distribution of right atrium according to sex in the population sample of GHS (n=9,511): values normalized for body surface area (BSA).

| **Right atrium** | **Mean** | **2SD-Interval** | **Median** | **5^th^-95^th^**  **Percentile** |
| --- | --- | --- | --- | --- |
| **Men** |  | | | |
| Circumference/BSA (cm/m²) | 7.56 | 6.02-9.10 | 7.52 | 6.35-8.88 |
| Area/BSA (cm²/m²) | 8.13 | 5.16-11.1 | 7.95 | 5.97-10.7 |
| Volume/BSA (ml/m²) | 22.2 | 9.20-35.2 | 21.3 | 13.4-34.3 |
| Septal-lateral diameter/BSA (cm/m²) | 1.85 | 1.36-2.34 | 1.83 | 1.47-2.28 |
| Apico-basal diameter/BSA (cm/m²) | 2.40 | 1.83-2.96 | 2.39 | 1.95-2.89 |
| **Women** |  | | | |
| Circumference/BSA (cm/m²) | 7.97 | 6.37-9.57 | 7.94 | 6.71-9.30 |
| Area/BSA (cm²/m²) | 7.84 | 5.16-10.5 | 7.70 | 5.93-10.2 |
| Volume/BSA (ml/m²) | 19.6 | 8.96-30.2 | 18.7 | 12.6-29.3 |
| Septal-lateral diameter/BSA (cm/m²) | 1.94 | 1.46-2.43 | 1.93 | 1.58-2.36 |
| Apico-basal diameter/BSA (cm/m²) | 2.54 | 1.96-3.12 | 2.53 | 2.08-3.02 |

Data presented are mean, the twofold standard deviation interval, the median and the 5^th^ to 95^th^ percentile interval.

**Table S5 of Supplement.** Distribution of right atrium according to sex in the reference sample of GHS (n=1,942): values normalized for body surface area BSA

| **Right atrium** | **Mean** | **2SD-Interval** | **Median** | **5^th^-95^th^**  **Percentile** |
| --- | --- | --- | --- | --- |
| **Men** |  | | | |
| Circumference/BSA (cm/m²) | 7.75 | 6.33-9.17 | 7.70 | 6.64-9.02 |
| Area/BSA (cm²/m²) | 8.41 | 5.56-11.3 | 8.24 | 6.29-11.1 |
| Volume/BSA (ml/m²) | 23.5 | 10.6–36.4 | 22.5 | 14.6-36.1 |
| Septal-lateral diameter/BSA (cm/m²) | 1.93 | 1.45-2.40 | 1.90 | 1.56-2.37 |
| Apico-basal diameter/BSA (cm/m²) | 2.44 | 1.92-2.96 | 2.42 | 2.02-2.91 |
| **Women** |  | | | |
| Circumference/BSA (cm/m²) | 8.10 | 6.60-9.59 | 8.05 | 6.97-9.37 |
| Area/BSA (cm²/m²) | 7.88 | 5.23-10.5 | 7.76 | 5.99-10.3 |
| Volume/BSA (ml/m²) | 19.5 | 9.09-30.0 | 18.8 | 12.6-28.7 |
| Septal-lateral diameter/BSA (cm/m²) | 2.00 | 1.53-2.46 | 1.98 | 1.65-2.42 |
| Apico-basal diameter/BSA (cm/m²) | 2.57 | 2.01-3.14 | 2.55 | 2.15-3.05 |

Data presented are mean, the twofold standard deviation interval, the median and the 5^th^ to 95^th^ percentile interval.

**Table S6 of Supplement.** Sex-specific associations between right atrial measurements and age in the GHS study samples.

β-estimates (ß) for age, coefficient of variation and skewness modelled for variables of the right atrium.

|  | **GHS population sample** | | | | | | | | | | |  | **Reference sample** | |
| --- | --- | --- | --- | --- | --- | --- | --- | --- | --- | --- | --- | --- | --- | --- |
| **Right atrium** | **Age (years)** | |  | | | **CV** | | | **Skewness** | | |  | **Age (years)** | |
|  | **ß** | **p** |  | | | **B-Estimate** | | **p** | **B-Estimate** | | **p** |  | **ß** | **p** |
| **Men** |  | | | | | | | | | | | | | |
| Circumference/Height (cm/m) | **1.46x10^-2^** | **<0.0001** | |  | | | **3.83x10^-4^** | **0.013** | 3.84x10^-3^ | | **0.66** |  | **1.29x10^-2^** | **<0.0001** |
| Area/Height (cm²/m) | **1.3x10^-2^** | **<0.0001** | |  | | | **1.12x10^-3^** | **0.00014** | **1.54x10^-2^** | | **0.0099** |  | **1.92x10^-2^** | **0.0019** |
| RA-volume/Height (ml/m) | 1.97x10^-2^ | 0.098 | |  | | | **1.84x10^-3^** | **<0.0001** | **1.48x10^-2^** | | **0.010** |  | 4.97x10^-2^ | 0.097 |
| Septal-lateral diameter/Height (cm/m) | 7.94x10^-4^ | 0.098 | |  | | | **1.84x10^-3^** | **<0.0001** | 5.29x10^-3^ | | 0.17 |  | 3.84x10^-4^ | 0.70 |
| Apico-basal diameter/Height (cm/m) | **6.83x10^-3^** | **<0.0001** | |  | | | **5.58x10^-4^** | **0.00079** | **7.77x10^-3^** | | **0.098** |  | **6.51x10^-3^** | **<0.0001** |
| **Women** |  | | | | | | | | | | | | | |
| Circumference/Height (cm/m) | **1.78x10^-2^** | **<0.0001** |  | | **3.38x10^-4^** | | | **0.0018** | | 6.72x10^-3^ | **0.13** |  | **1.70x10^-2^** | **<0.0001** |
| Area/Height (cm²/m) | **1.77x10^-2^** | **<0.0001** |  | | **6.79x10^-4^** | | | **0.0013** | | **1.27x10^-2^** | **0.023** |  | **1.67x10^-2^** | **0.00074** |
| RA-volume/Height (ml/m) | **3.24x10^-2^** | **0.00056** |  | | **1.07x10^-3^** | | | **0.0021** | | **1.62x10^-2^** | **0.011** |  | 3.40x10^-2^ | 0.059 |
| Septal-lateral diameter/Height (cm/m) | **1.97x10^-3^** | **<0.0001** |  | | **1.87x10^-4^** | | | **0.11** | | **1.75x10^-3^** | **0.67** |  | **2.70x10^-3^** | **0.00052** |
| Apico-basal diameter/Height (cm/m) | **6.71x10^-3^** | **<0.0001** |  | | **5.1x10^-4^** | | | **0.0010** | | **4.99x10^-3^** | **0.22** |  | **8.09x10^-3^** | **<0.0001** |

**Figure S1 of Supplement.** Sex-specific nomogram for circumference/height of the right atrium stratified for age. The lines mark the 95% percentile of reference sample (<95%) and 98^th^ and 99^th^ percentiles of the GHS population sample. <95% of reference sample is the normal reference (green area). Mild deviation is marked in yellow, severe deviation in orange and very severe deviation in red.


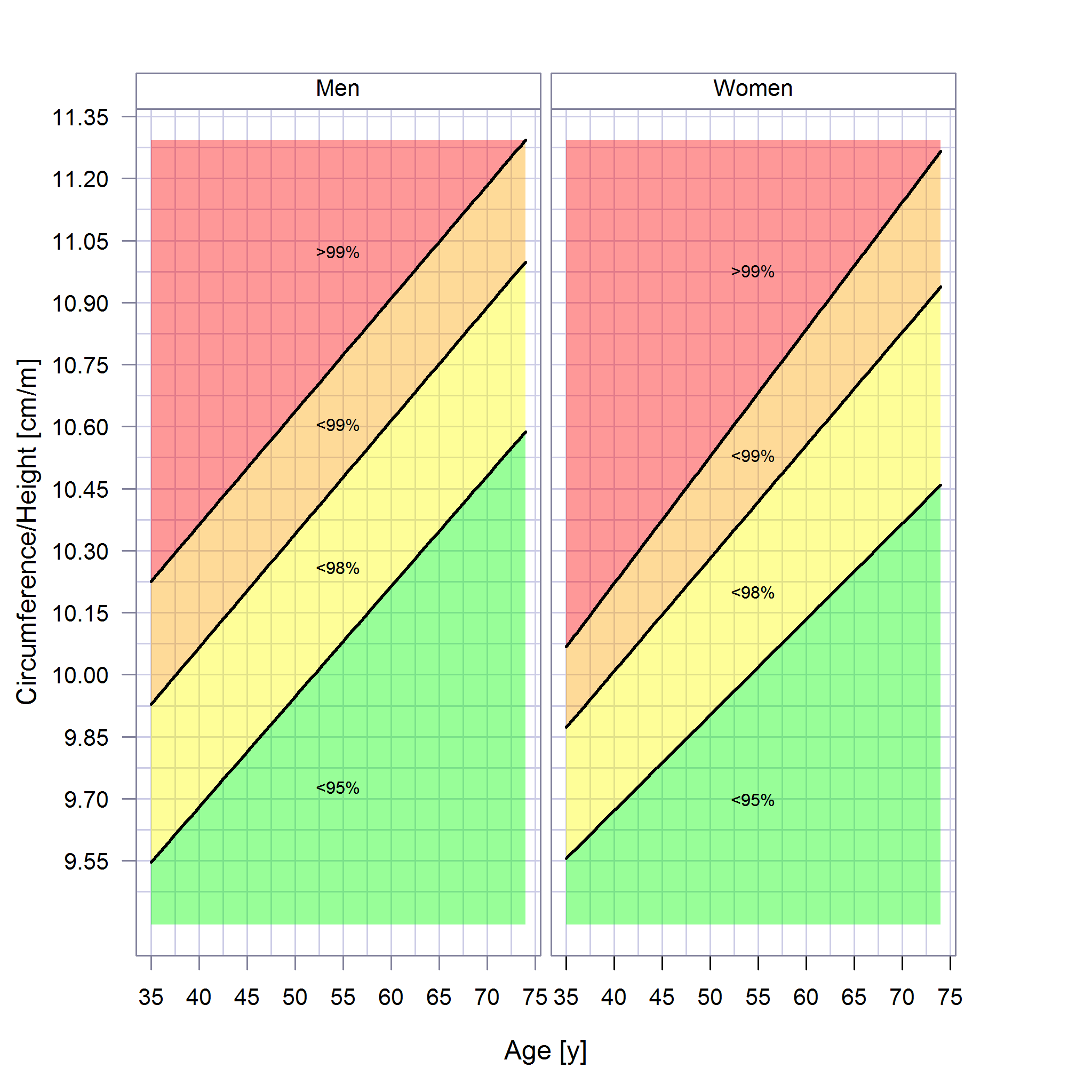


**Figure S2 of Supplement.** Sex-specific nomogram for area/height of the right atrium stratified for age. The lines mark the 95% percentile of reference sample (<95%) and 98^th^ and 99^th^ percentiles of the GHS population sample. <95% of reference sample is the normal reference (green area). Mild deviation is marked in yellow, severe deviation in orange and very severe deviation in red.


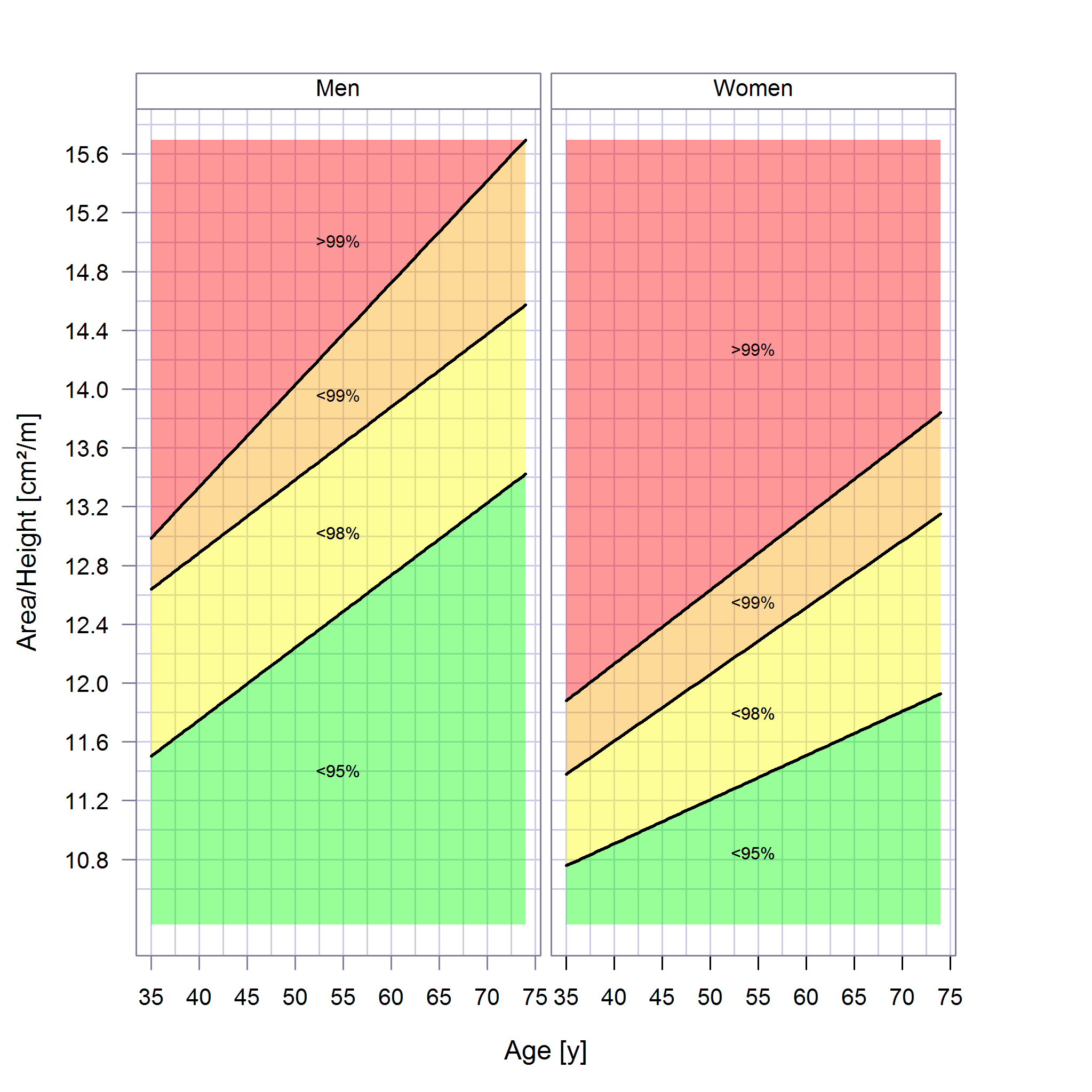


**Figure S3 of Supplement.** Sex-specific nomogram for septal-lateral diameter/height of the right atrium stratified for age. The lines mark the 95% percentile of reference sample (<95%) and 98^th^ and 99^th^ percentiles of the GHS population sample. <95% of reference sample is the normal reference (green area). Mild deviation is marked in yellow, severe deviation in orange and very severe deviation in red.


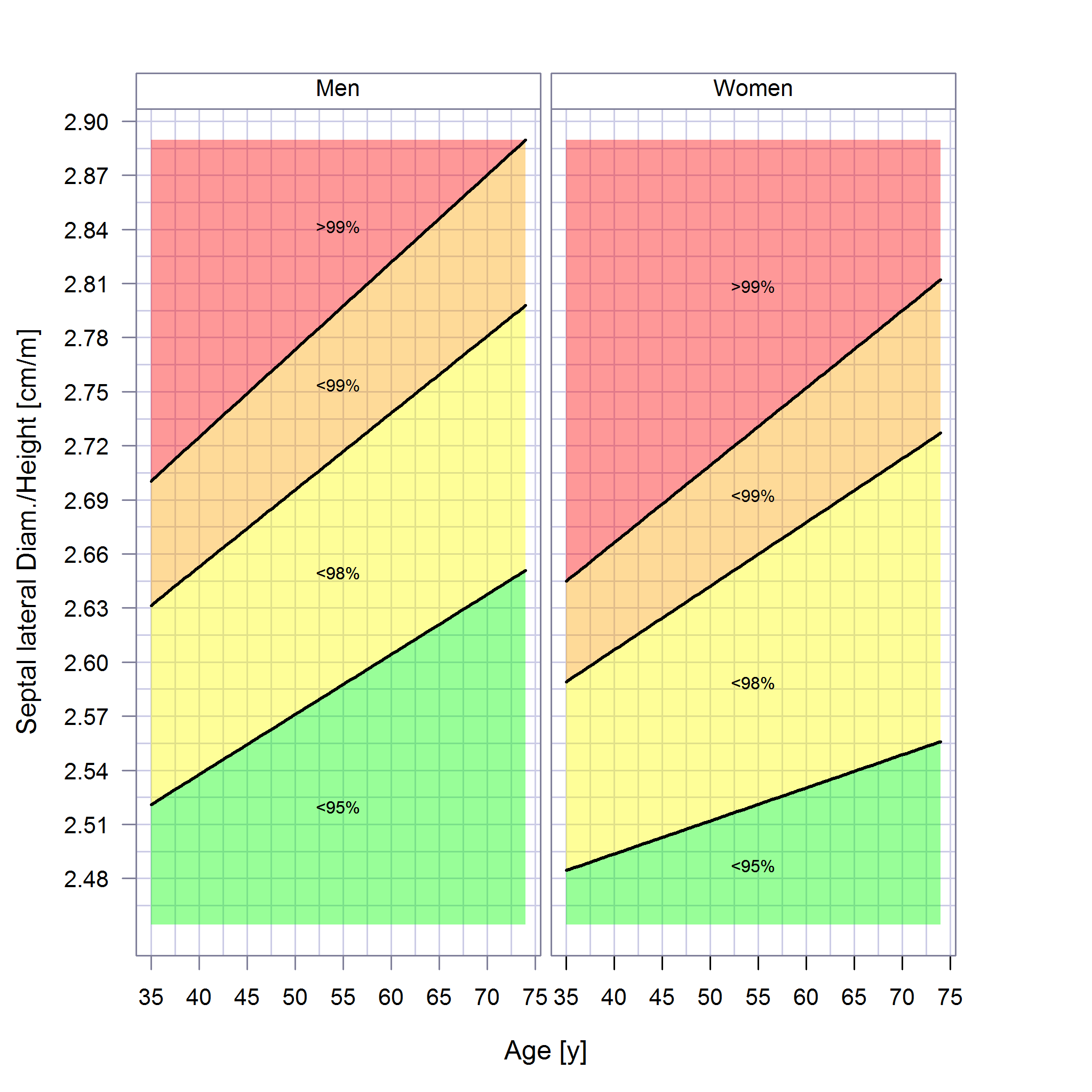


**Figure S4 of Supplement.** Sex-specific nomogram for apico-basal diameter/height of the right atrium stratified for age. The lines mark the 95% percentile of reference sample (<95%) and 98^th^ and 99^th^ percentiles of the GHS population sample. <95% of reference sample is the normal reference (green area). Mild deviation is marked in yellow, severe deviation in orange and very severe deviation in red.


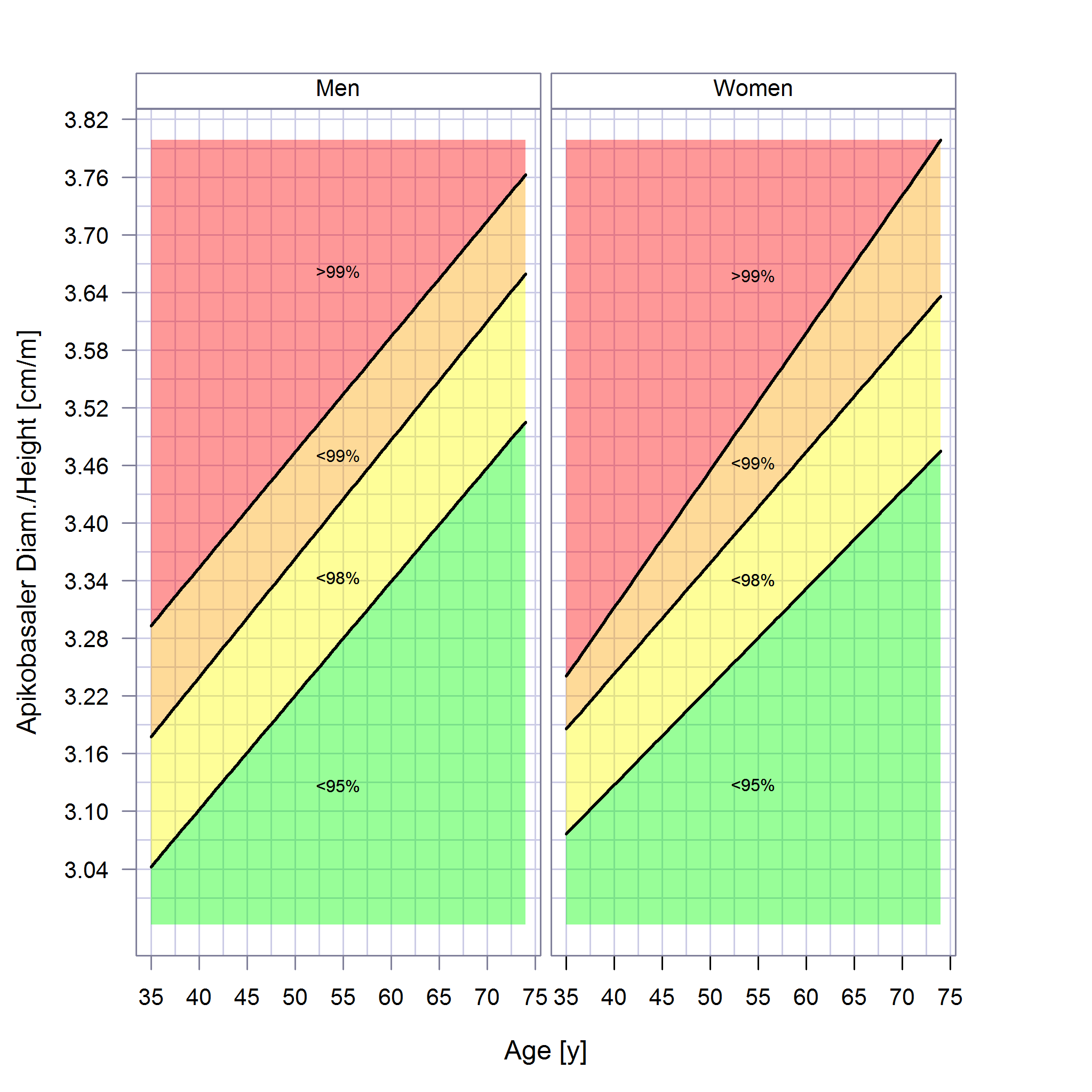

Supplement: Supplementary file 1 — Supplementary Information. [file 41598_2021_1968_MOESM1_ESM.docx]
